# Beyond the Operating Room? Cytology-Based Yield and Resource Utilization of Outpatient Versus Operating Room EBUS-TBNA Pathways

**DOI:** 10.3390/diagnostics16172767

**Published:** 2026-08-28

**Authors:** Paolo Albino Ferrari, Cosimo Bruno Salis, Sabrina Sarais, Massimiliano Santoru, Matteo Pinna-Susnik, Sonia Nemolato, Fabiola Farci, Elisabetta Pusceddu, Alessandro Tamburrini, Oliver Harrison, Francesco Guerrera, Paraskevas Lyberis, Daniela Ledda, Graziano Carta, Laura Riva, Giuseppe Porcu, Stefano Marini, Paolo Siotto, Alessandro Giuseppe Fois, Antonio Macciò

**Affiliations:** 1 Department of Thoracic Surgery, Azienda di Rilievo Nazionale ed Alta Specializzazione “G. Brotzu”, Piazza A. Ricchi 1, 09121 Cagliari, Italy; sabrina.sarais@aob.it (S.S.); massimiliano.santoru@aob.it (M.S.); matteo.pinnasusnik@aob.it (M.P.-S.); laura.riva@aob.it (L.R.); 2 Department of Oncological Surgery, Azienda di Rilievo Nazionale ed Alta Specializzazione “G. Brotzu”, Piazza A. Ricchi 1, 09121 Cagliari, Italy; daniela.ledda@aob.it (D.L.); grziano.carta@aob.it (G.C.); antoniopm.maccio@aob.it (A.M.); 3 Department of Medicine, Surgery and Pharmacology, University of Sassari, Viale San Pietro 43, 07100 Sassari, Italy; cosimobrunosalis@gmail.com; 4 Unit of Anatomic Pathology, Azienda di Rilievo Nazionale ed Alta Specializzazione “G. Brotzu”, Piazza A. Ricchi 1, 09121 Cagliari, Italy; sonia.nemolato@aob.it (S.N.); fabiola.farci@aob.it (F.F.); giuseppes.porcu@aob.it (G.P.); 5 Anesthesia and Intensive Care Unit, Liver Transplantation Center, Azienda di Rilievo Nazionale ed Alta Specializzazione “G. Brotzu”, Piazza A. Ricchi 1, 09121 Cagliari, Italy; elisabetta.pusceddu@aob.it; 6 Department of Thoracic Surgery of Wessex Cardiothoracic Center, University Hospital Southampton NHS Foundation Trust, Southampton SO16 6YD, UK; alessandro.tamburrini@uhs.nhs.uk (A.T.); oliver.harrison@uhs.nhs.uk (O.H.); 7 Department of Thoracic Surgery, Azienda Ospedaliero Universitaria Città della Salute e della Scienza di Torino, Corso Bramante 88, 10126 Turin, Italy; francesco.guerrera@unito.it (F.G.); paraskevas.lyberis@unito.it (P.L.); 8 Anesthesia Unit, “A. Businco” Oncology Hospital, via Jenner 8, 09121, Cagliari, Italy; 9 Departmental Unit of Oncologic Radiology, “A. Businco” Oncology Hospital, via Jenner 8, 09121 Cagliari, Italy; stefano.marini@aob.it; 10 Department of Radiology, Azienda di Rilievo Nazionale ed Alta Specializzazione “G. Brotzu”, Piazza A. Ricchi 1, 09121 Cagliari, Italy; paolo.siotto@aob.it; 11 Clinical and Interventional Respiratory Unit, University Hospital of Sassari (AOU), Viale San Pietro 43, 07100 Sassari, Italy; agfois@uniss.it

**Keywords:** endobronchial ultrasound (EBUS), transbronchial needle aspiration (TBNA), cytology-based yield, outpatient pathway, operating room pathway, sedation, general anesthesia, resource utilization, cost analysis

## Abstract

**Background/Objectives:** Endobronchial ultrasound-guided transbronchial needle aspiration (EBUS-TBNA) can be performed via pathways that differ in anesthesia, setting, airway management, monitoring, recovery, and hospitalization. We compared an outpatient endoscopy suite pathway with sedation and spontaneous breathing, and an operating room pathway with general anesthesia and laryngeal mask ventilation, assessing cytology-based yield, sample adequacy, workflow, and costs. **Methods:** This single-center observational study included consecutive EBUS-TBNA procedures. Allocation was nonrandom and reflected workflow, operating room availability, complexity, airway/anesthetic considerations, and judgment. The primary endpoint was cytology-based diagnostic yield in the diagnostic cohort; staging outcomes included cytology distribution, sample adequacy, and positivity among adequate samples. Systematic diagnostic verification was not available for the entire cohort; however, postoperative nodal pathology was retrospectively retrieved when available in the staging cohort. Because verification remained incomplete and selectively available, sensitivity, specificity, negative predictive value, and overall diagnostic accuracy were not estimated. **Results:** Clinical outcome analyses included 447 procedures (319 diagnostic and 128 staging), while the administrative cost dataset included 533 completed procedures. Diagnostic yield was similar between pathways (108/229, 47.2% vs. 45/90, 50.0%; *p* = 0.740). In the staging cohort, sample adequacy was comparable (83/85, 97.6% vs. 39/42, 92.9%; *p* = 0.331), and positivity among adequate samples did not differ (21.7% vs. 20.5%; *p* = 1.000). Exploratory postoperative pathological nodal-stage data were available for 60/128 staging procedures; among 58 cases with interpretable EBUS-TBNA results, patient-level N-stage concordance was 44/58 (75.9%), and postoperative nodal upstaging occurred in 11/58 (19.0%). No prespecified intraprocedural composite adverse events were documented. Duration appeared shorter in operating room cases but, owing to incomplete differential recording, was descriptive only. Length of stay and costs were markedly higher in the operating room pathway. **Conclusions:** The pathways showed similar cytology-based outcomes but differed in hospitalization rates and costs. Nonrandom allocation and pathway heterogeneity preclude causal interpretation as a sedation-versus-general-anesthesia comparison. Prospective studies with standardized allocation, diagnostic verification, systematic capture of adverse events, and follow-up are warranted.

## 1. Introduction

Endobronchial ultrasound-guided transbronchial needle aspiration (EBUS-TBNA) is a cornerstone minimally invasive procedure for diagnosing and staging mediastinal and hilar lymphadenopathy in suspected lung cancer and selected benign conditions, including granulomatous and lymphoproliferative diseases [1,2,3,4,5,6,7]. EBUS-TBNA is performed under heterogeneous anesthetic strategies, ranging from moderate or deep sedation in a bronchoscopy/endoscopy suite to general anesthesia (GA) with assisted ventilation in the operating room (OR) [8,9,10,11,12,13,14,15,16,17].

Available comparative evidence is mixed: a randomized trial reported similar diagnostic yield under GA versus moderate sedation, despite higher procedural completion and fewer minor events under GA, whereas a multicenter prospective study found no meaningful differences in adequacy/accuracy between intravenous GA with neuromuscular blockade and intravenous sedation with topical anesthesia [8,10]. Conversely, sedation depth may influence sampling intensity and yield in suite-based practice, with higher yield under deep versus moderate sedation in some cohorts, but not consistently across studies [9,13,18].

Although previous studies have generally reported comparable cytology-based diagnostic yield between sedation and general anesthesia strategies, direct comparisons of resource utilization between outpatient endoscopy suite and operating room EBUS-TBNA pathways remain limited. In particular, itemized cost analyses separating hospitalization-related and non-hospitalization procedural costs are sparse. Against this background, we conducted a single-center observational study comparing two institutional EBUS-TBNA care pathways—an outpatient endoscopy suite pathway under sedation with spontaneous breathing and an operating room pathway under general anesthesia with laryngeal mask airway ventilation—with respect to cytology-based yield, sample adequacy, workflow, and economic outcomes [10,13,14,19,20,21].

More broadly, this study was designed to determine whether EBUS-TBNA should be considered a procedure routinely dependent on operating room and anesthesiology-based pathways, or whether an outpatient endoscopy suite pathway may provide a clinically and organizationally viable option for appropriately selected patients.

## 2. Materials and Methods

This single-center observational comparative study was conducted at the Thoracic Surgery and Interventional Thoracic Endoscopy Unit (A.R.N.A.S. “G. Brotzu”, Cagliari, Italy), comparing two institutional EBUS-TBNA care pathways rather than isolated anesthesia modalities. The outpatient pathway consisted of EBUS-TBNA performed in an endoscopy suite under sedation with topical anesthesia and spontaneous breathing, followed by short-term recovery monitoring and same-day discharge. The operating room pathway consisted of EBUS-TBNA performed under general anesthesia with laryngeal mask airway ventilation, operating room monitoring and anesthesiology support, followed by routine post-procedural hospitalization. Accordingly, the exposure of interest bundled procedural setting, airway and ventilation strategy, monitoring environment, recovery logistics, and hospitalization policy.

The study was conducted in accordance with the Declaration of Helsinki and approved by the Territorial Ethics Committee, Regional Ethics Committee of Sardinia, Italy (Ethics Committee Minutes No. 03/2026, meeting held on 9 June 2026). Written informed consent for study participation and for the processing of personal data for research purposes was distributed to and signed by all participants before inclusion in the research dataset.

The primary endpoint was cytology-based diagnostic yield in the diagnostic cohort, defined as the proportion of procedures resulting in cytology considered diagnostic for the clinical question. This endpoint should be distinguished from diagnostic accuracy. In the staging cohort, cytology was categorized as negative, positive for neoplastic cells, or not adequate, with adequacy assessed using a binary definition (adequate = negative/positive; not adequate = insufficient or unsuitable material). For the staging cohort, an additional retrospective postoperative pathological ascertainment was performed. Electronic clinical and pathological records were reviewed to retrieve postoperative pathological nodal stage (pN/ypN, as applicable) and involved nodal stations in patients who subsequently underwent surgical resection and for whom postoperative pathology records were available. EBUS-derived nodal stage was compared descriptively with postoperative pathological nodal stage at the patient level and, in cases of postoperative nodal upstaging, sampled EBUS stations were compared with surgically involved nodal stations.

For this exploratory analysis, EBUS-derived N stage was assigned according to the highest nodal category represented by a cytology-positive sampled station. In interpretable examinations without a cytology-positive sampled station, EBUS-derived nodal status was categorized as N0; examinations with an overall not adequate EBUS-TBNA result were excluded from N-stage concordance assessment.

Patients with documented neoadjuvant treatment occurring after EBUS-TBNA and before surgery were not interpreted as direct discordances between EBUS-positive findings and postoperative pathological N0 status. Because postoperative verification was selectively available only in a surgical subset, this analysis was considered exploratory and was not used to estimate sensitivity, specificity, negative predictive value, or overall diagnostic accuracy.

Secondary outcomes included procedure duration (exploratory, given differential recording completeness), LOS (including LOS > 0), staging cohort cytology distribution, sample adequacy and positivity among adequate samples, the distribution of diagnostic suspicion and cytologic diagnostic categories, the association between suspicion profile and procedural setting, nodal sampling characteristics (stations sampled, positron emission tomography (PET) positivity documentation and rapid on-site evaluation (ROSE) utilization), exploratory associations between sampling strategy (station sampled yes/no, number of stations sampled, and station combination patterns) and cytology adequacy in the staging cohort, and economic outcomes (total costs and components), including the contribution of LOS and the independent association of setting and LOS with total costs. No systematic post-procedural clinical follow-up outcomes were prespecified or collected for the main study cohorts; the retrospective postoperative pathological ascertainment described above was performed solely as an exploratory analysis in the staging subset.

Adverse events were retrospectively captured from the endoscopy report and, for operating room procedures, from the anesthesiology chart. The prespecified intraprocedural composite included bleeding requiring intervention, sustained oxygen desaturation requiring escalation, hemodynamic instability requiring pharmacologic treatment, and unplanned airway or ventilation escalation. Minor self-limited events, cough, transient desaturation not requiring escalation, patient discomfort, delayed complications, post-discharge symptoms, and patient-reported outcomes were not systematically captured.

All procedures were performed in a supervised setting, with a senior thoracic surgery consultant (with >250 EBUS procedures) directly overseeing all procedural steps. Allocation to the outpatient endoscopy suite pathway or to the operating room pathway was not randomized and reflected real-world institutional decision-making. This included operating room availability, procedural scheduling, anticipated technical complexity, airway and anesthetic considerations, patient-level clinical factors, peri-procedural logistics, and senior consultant judgment. Therefore, the study was not designed to estimate the independent causal effect of anesthesia modality or procedural setting, and residual confounding by indication should be expected.

For both diagnostic and staging EBUS, the pre-procedural work-up followed an institutional pathway including cardiovascular risk stratification for the planned intervention (cardiology consultation and further investigations when indicated), a standardized blood test panel (complete blood count, coagulation profile, and liver and renal function tests), and thoracic imaging with contrast-enhanced chest computed tomography (CT); positron emission tomography/computed tomography (PET/CT) with 18F-fluorodeoxyglucose (18F-FDG) was performed when required by the clinical scenario. For patients scheduled for operating room procedures, a dedicated pre-procedural anesthesiology assessment was also routinely performed.

In the outpatient endoscopy suite (Level I endoscopic outpatient setting compliant with Italian regulations), patients were positioned on a reclining chair in a supine/semi-recumbent posture, with the operator positioned at the head of the patient. A peripheral intravenous line was placed, and airway patency was maintained with a mouthpiece. Vital signs monitoring included non-invasive blood pressure, peripheral oxygen saturation, and heart rate. Sedation was titrated according to institutional practice toward a clinically appropriate moderate sedation target on a standardized scale (e.g., Ramsay Sedation Scale 3–4 or Observer’s Assessment of Alertness/Sedation 3–4). However, sedation depth scores were not systematically recorded as analyzable case-level variables. Supplemental oxygen was delivered via nasal cannula or facial mask, titrated up to 12 L/min to maintain adequate oxygenation. Capnography was not available in the outpatient endoscopy suite; therefore, monitoring relied on non-invasive blood pressure, heart rate, and peripheral oxygen saturation. Topical anesthesia with lidocaine was administered within institutional safety limits (maximum cumulative dose according to local protocol, weight-adjusted).

In the operating room, patients were supine on an operating table, with the operator positioned at the head of the patient. Monitoring included ECG, non-invasive blood pressure, and SpO_2_, with a peripheral intravenous line for drug administration. General anesthesia was delivered using a total intravenous anesthesia/target-controlled infusion (TIVA/TCI) protocol (propofol with fentanyl and/or remifentanil) with neuromuscular blockade. Airway management was achieved with positive pressure ventilatory support via a cuffed laryngeal mask airway (LMA) by INTERSURGICAL Ltd. (Wokingham, Berkshire, UK).

All procedures used a PENTAX Medical (Tokyo, Japan) EB19-J10^®^ echo-bronchoscope with a latest-generation PENTAX Medical^®^ video bronchoscopy platform and a FUJIFILM Corporation (Tokyo, Japan) Arietta 750^®^ ultrasound system. For both diagnostic and staging indications, only COOK MEDICAL LLC (Bloomington, IN, USA) EchoTip Ultra^®^ 22G endobronchial ultrasound HD needles were used, with a protocol-mandated minimum of three passes per sampled nodal station [22].

When available according to Pathology Service scheduling, rapid on-site evaluation (ROSE) was performed by a pathologist to assess specimen adequacy. Material was smeared onto slides for ROSE, and a residual sample was collected in HOLOGIC (Zaventem, Belgium) ThinPrep™-CytoLyt™ solution for cytology processing/cell block preparation. ROSE utilization was not randomized and depended on Pathology Service scheduling and availability. Therefore, ROSE was analyzed as a descriptive procedural variable rather than as a controlled intervention.

After outpatient procedures, patients underwent 3 h of clinical monitoring in a recovery area before discharge. In contrast, after operating room procedures, patients generally remained hospitalized for at least one overnight stay in the thoracic surgery ward.

### Statistical Analysis

Continuous variables are reported as mean ± standard deviation (SD) and median interquartile range (IQR), and categorical variables as *n* (%). Group comparisons used Welch’s *t*-test and/or Mann–Whitney U test for continuous variables and chi-square or Fisher’s exact test for categorical variables, as appropriate; the association between procedural setting and diagnostic suspicion was assessed by chi-square with Cramér’s V.

In the diagnostic cohort, diagnostic yield was analyzed overall and by suspicion strata. Exploratory complete-case logistic regression including recorded procedure duration was not used to support primary conclusions because duration recording differed substantially across pathways.

In the staging cohort, cytology was analyzed as negative/positive/not adequate and, for adequacy analyses, dichotomized as adequate (negative/positive) vs. not adequate; exploratory associations with nodal sampling strategy (station sampled, number of stations, and station combination patterns) were evaluated with Fisher’s exact/nonparametric tests and supportive univariate logistic modeling (interpreted cautiously given the low frequency of not adequate results). The postoperative pathological ascertainment analysis was descriptive. Patient-level EBUS-derived N stage was compared with postoperative pathological nodal stage, and postoperative upstaging cases were additionally reviewed at the nodal station level. No inferential comparison or diagnostic accuracy estimates were performed because postoperative verification was selectively available and did not constitute a complete reference standard for the entire staging cohort.

Procedure duration was not uniformly recorded across settings; therefore, analyses involving duration were performed using complete-case data only. No imputation was performed because missingness was not assumed to be random and differed by pathway; duration analyses were therefore considered exploratory.

Economic analyses used the broader administrative dataset of completed EBUS-TBNA procedures with valid cost information, including procedures excluded from the clinical outcome cohorts because of missing key clinical variables. Cost analyses compared total costs by setting and procedure type nonparametrically and used a Gamma generalized linear model (log link) including LOS, with robust linear regression as a sensitivity analysis.

Analyses were performed using Stata/MP 13.1 (StataCorp, College Station, TX, USA). ChatGPT-5.4 (OpenAI) supported investigator-supervised auditing for missingness and data plausibility before modeling. All tests were two-sided with *p* < 0.05 considered statistically significant.

## 3. Results

A total of 447 EBUS-TBNA procedures were included across two clinical cohorts: a diagnostic cohort (*n* = 319) and a staging cohort (*n* = 128). Baseline demographics were broadly comparable across procedural settings within each cohort, with no clinically relevant differences in age distribution or sex composition (Table 1). However, several selection-related clinical variables were not systematically available in the retrospective dataset, including ASA physical status, BMI, COPD or obstructive sleep apnea, baseline oxygen requirement, anticoagulant or antiplatelet therapy, lesion or lymph node size, prior nondiagnostic bronchoscopy, and the need for additional concurrent procedures. In contrast, workflow-related parameters differed markedly.

In both cohorts, procedures performed in the operating room pathway were associated with shorter recorded procedure duration and higher length of stay (LOS) compared with procedures performed in the outpatient endoscopy suite pathway (Table 1). However, this finding should be interpreted as exploratory, as duration availability varied substantially across settings, with higher completeness in operating room cases. Therefore, recorded duration may reflect documentation practices and case selection patterns in addition to true procedural time. No prespecified intraprocedural composite adverse events were documented in either pathway, including bleeding requiring intervention, sustained desaturation requiring escalation, hemodynamic instability requiring treatment, or unplanned airway/ventilation escalation. In addition, no unplanned conversion from the outpatient endoscopy suite pathway to the operating room pathway was documented.

However, systematic post-discharge follow-up was not available; therefore, delayed or minor post-procedural adverse events could not be reliably assessed.

**Figure 1:**
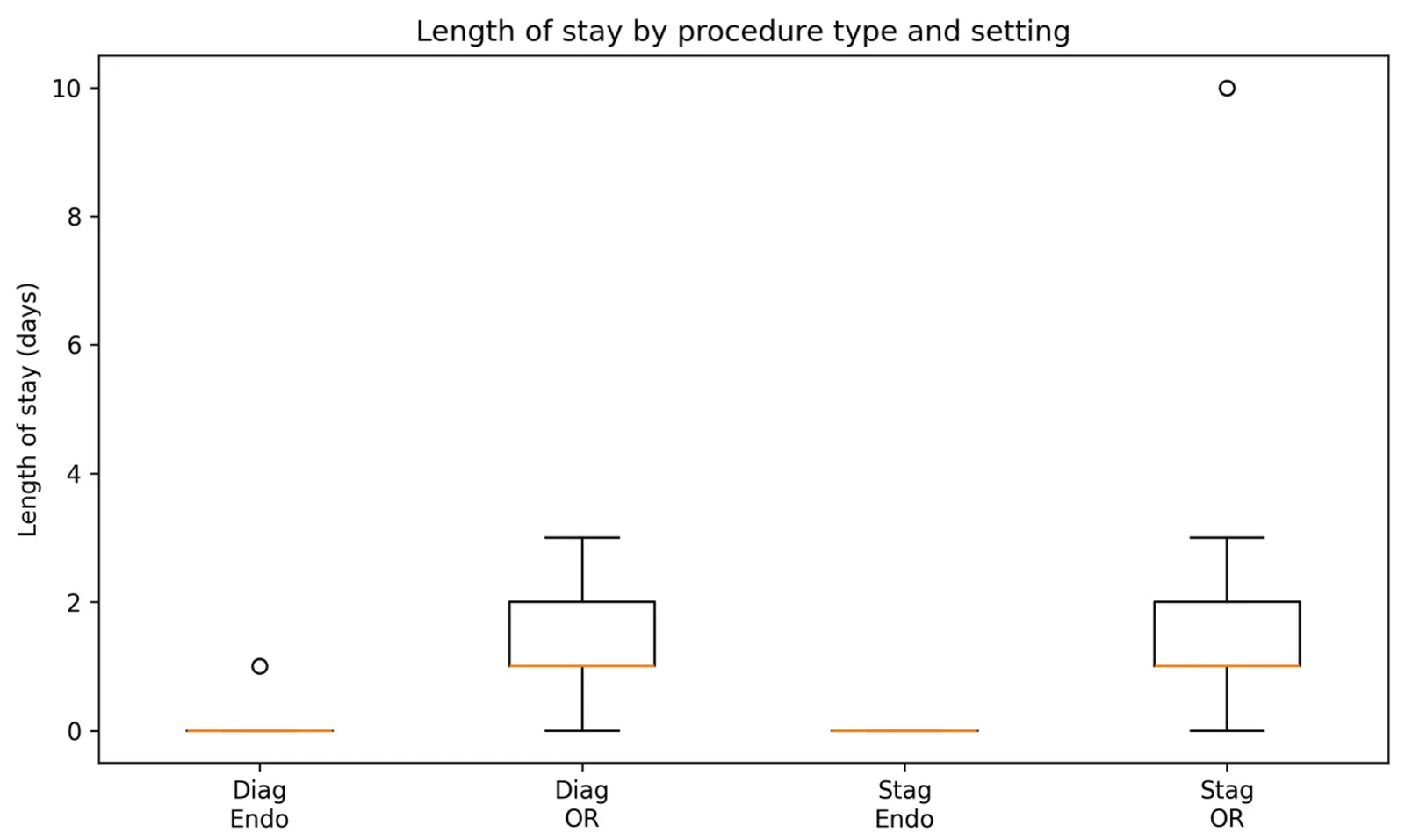
Length of stay (LOS, days) by procedure type and procedural setting. Boxplots show median and interquartile range with whiskers. LOS was concentrated in the operating room pathway, whereas LOS was almost uniformly 0 days in the outpatient endoscopic room pathway, with only one outpatient case having LOS = 1 day. Abbreviations: Diag, diagnostic EBUS; Endo, endoscopic room; Stag, staging EBUS; OR, operating room.

Prolonged hospital stays were confined to the operating room pathway: no outpatient endoscopy suite procedure resulted in LOS > 1 day. One outpatient endoscopy suite case had LOS = 1 day, whereas no outpatient procedure exceeded 1 day of hospitalization. Among operating room cases, three patients had an LOS of 3 days due to non-clinical, family/organizational reasons. In contrast, one outlier stay of 31 days was attributable to interdisciplinary oncologic management during hospitalization (treatment delivered for recurrent adenocarcinoma), rather than procedure-related complications. Notably, the single 31-day hospitalization was retained in the statistical analyses; a sensitivity check excluding this case confirmed that the overall findings were unchanged, with differences limited to the upper range (maximum LOS). The pronounced separation in hospitalization patterns across settings is also summarized visually in Figure 1.

### 3.1. Nodal Sampling Patterns, PET Positivity, and ROSE Utilization

Nodal sampling patterns were consistent with routine mediastinal assessment. Station 7 was the most frequently sampled in both cohorts, followed by the right paratracheal and hilar stations, whereas other stations were sampled less frequently (Table 2).

Station-level documentation of PET positivity was variably available across stations and cohorts, whereas ROSE utilization was more consistently captured, enabling a descriptive overview of ROSE implementation among sampled nodes (Table 2). ROSE availability was not randomized and depended on Pathology Service scheduling; therefore, it should be interpreted as a potential procedural confounder rather than as a controlled exposure.

To further evaluate whether ROSE utilization differed across institutional pathways, procedure-level and station-level ROSE use were stratified by cohort and procedural setting (Table S1). Procedure-level ROSE utilization was comparable between the outpatient endoscopy suite and operating room pathways in both the diagnostic cohort (161/228, 70.6% vs. 64/89, 71.9%; Fisher’s exact *p* = 0.891) and the staging cohort (62/85, 72.9% vs. 30/42, 71.4%; Fisher’s exact *p* = 1.000).

In the staging cohort, sampling strategies commonly involved single-station sampling (most frequently station 7) and, less often, multi-station combinations such as 4R + 7 or 7 + 10R, consistent with the most prevalent sampling patterns (Table S2). These descriptive data provide context for subsequent analyses evaluating cytology adequacy in relation to sampling extent and patterns (Tables S3 and S4).

Within the diagnostic cohort, diagnostic yield varied numerically across suspicion categories, with the highest proportion of diagnostic cytology observed in suspected sarcoidosis; however, differences in diagnostic yield across the three main suspicion groups did not reach statistical significance. In this context, a negative cytology result was interpreted as adequate sampling without suspicious cellularity, whereas an inadequate result reflected insufficient material precluding a definitive diagnosis (Table S5).

### 3.2. Diagnostic Cohort: Diagnostic Suspicion, Cytology Spectrum, Diagnostic Yield, and Agreement Metrics

In the diagnostic cohort, clinical suspicion was predominantly coded as suspected malignancy, with smaller proportions coded as sarcoidosis and lymphoproliferative disease; a limited fraction of records lacked a suspicion code (Table S6). The distribution of diagnostic suspicion categories was broadly comparable across procedural settings, with no statistically significant association between suspicion profile and setting (Table S7).

The cytology spectrum showed a predominance of negative results. At the same time, among malignant diagnoses, adenocarcinoma (ADC) and squamous cell carcinoma (SCC) were the most frequent primary lung cancer subtypes, with additional categories including metastases, sarcoidosis, and lymphoproliferative disease (Table S6).

Despite these distributions, the binary diagnostic yield (diagnostic vs. nondiagnostic cytology) did not differ meaningfully across procedural settings (Table 3).

As an illustrative example of diagnostic cytology obtained via EBUS-TBNA, representative cytomorphological and immunocytochemical findings from a case of small-cell lung carcinoma are shown in Figure S1.

Within the limits of cytology-based endpoints, this observation remained consistent in unadjusted comparisons and suspicion-stratified analyses. Agreement between the clinical suspicion category and the cytology-derived diagnostic category was low to fair overall, and concordance/κ metrics did not support meaningful differences between settings (Table 3). These analyses do not represent diagnostic accuracy estimates because final diagnosis adjudication and systematic follow-up were not available.

### 3.3. Staging Cohort: Cytology Categories, Adequacy, and Positivity

In the staging cohort, baseline histology was predominantly SCC, followed by ADC, with smaller proportions of other primary lung cancers and pulmonary metastases (Table S8). Cytology was evaluated using a three-level outcome (negative, positive for neoplastic cells, or not adequate), with negative results representing the largest category and not adequate samples being uncommon (Table S8). The distribution of cytology categories was broadly similar across settings, and both sample adequacy (negative/positive vs. not adequate) and positivity among adequate samples did not differ materially (Table 3).

To explore whether sampling strategy could influence cytology adequacy, we assessed adequacy in relation to both the extent of nodal sampling and sampling patterns. The number of sampled nodal stations per procedure clustered around one to two stations, with progressively fewer procedures sampling three or more stations (Table S3). Across these strata, inadequate cytology remained infrequent and showed no evidence of a systematic association with the number of sampled stations (Tables S3 and S4).

Consistently, an exploratory analysis of common station combination patterns did not reveal a reproducible pattern-specific enrichment of not adequate results. However, interpretation is constrained by the low event count for not adequate cytology (Table S2). In a station-level exploratory analysis (sampled vs. not sampled for each nodal station), no station showed a reproducible association with not adequate cytology. However, interpretation is limited by the low number of not adequate cases (Table S9).

Overall, these analyses suggest that, within this staging dataset, cytology adequacy was not meaningfully associated with either the number of sampled stations or the predominant sampling patterns, while acknowledging limited power due to the rarity of not adequate outcomes (Tables S3 and S4).

### 3.4. Exploratory Postoperative Nodal Pathology Ascertainment in the Staging Cohort

Postoperative pathological nodal stage could be retrospectively retrieved for 60/128 staging EBUS-TBNA procedures (46.9%). Of these, 21 procedures had been performed in the operating room pathway and 39 in the outpatient endoscopy suite pathway. Two cases, one from each pathway, had an overall non-adequate EBUS-TBNA result and were excluded from N-stage concordance assessment, leaving 58 interpretable comparisons.

At the patient level, EBUS-derived and postoperative pathological N stage were concordant in 44/58 cases (75.9%). Postoperative nodal upstaging occurred in 11/58 cases (19.0%). Three EBUS-positive cases subsequently had postoperative pathological N0 status. Two of these patients had documented neoadjuvant treatment after EBUS-TBNA and before surgery and were therefore not interpreted as evidence of false positive EBUS-TBNA; interval treatment information was unavailable for the remaining case.

Station-level review of the 11 postoperative upstaging cases showed that in 7/11 cases (63.6%), postoperative nodal involvement occurred in stations that had not been sampled during the index EBUS-TBNA, including cases involving stations 5 and/or 6, which were not routinely sampled through the institutional EBUS pathway. In the remaining 4/11 cases (36.4%), a nodal station previously sampled with negative EBUS-TBNA cytology was subsequently found to be involved at postoperative pathological examination. In one of these four cases, EBUS-TBNA had already identified N1 disease, whereas postoperative pathology demonstrated additional N2 involvement at a previously negative station 7; this therefore represented nodal understaging rather than an overall false negative EBUS-TBNA examination. Detailed findings are reported in Table S10.

### 3.5. Cost Analysis and the Role of Length of Stay

Cost data were available for 533 procedures (Table 4). The analyses used the full administrative dataset of completed EBUS procedures, including cases excluded from diagnostic outcome analyses for missing key clinical variables, because cost information remained valid for completed procedures.

**Figure 2:**
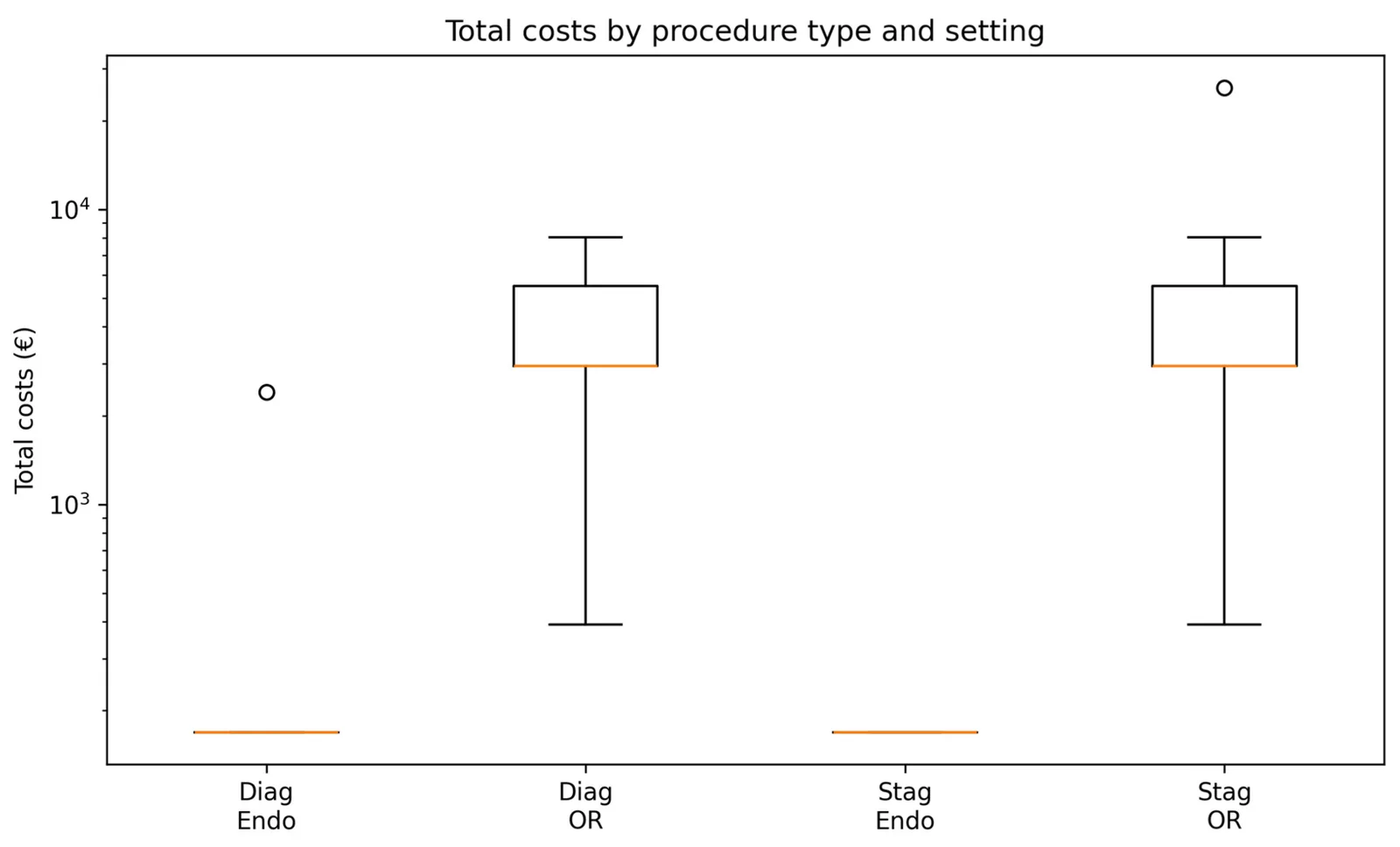
Total costs (€) by procedure type and procedural setting. Boxplots show median and interquartile range with whiskers. Operating room procedures had higher total costs than endoscopy suite procedures overall (Mann–Whitney U: *p* < 0.0001) and within both diagnostic (*p* < 0.0001) and staging pathways (*p* < 0.0001). Within the operating room, staging procedures showed higher total costs than diagnostic procedures (*p* = 0.008), whereas the difference between procedure types in the endoscopy suite was smaller (*p* = 0.563). Abbreviations: Diag, diagnostic EBUS; Endo, endoscopy suite; Stag, staging EBUS; OR, operating room.

Total costs were highly skewed and were largely driven by hospitalization-related components. In descriptive and nonparametric comparisons, procedures performed in the operating room were associated with substantially higher total costs than those performed in the endoscopy suite across both diagnostic and staging procedures (Table 4 and Table 5 and Figure 2).

**Figure 3:**
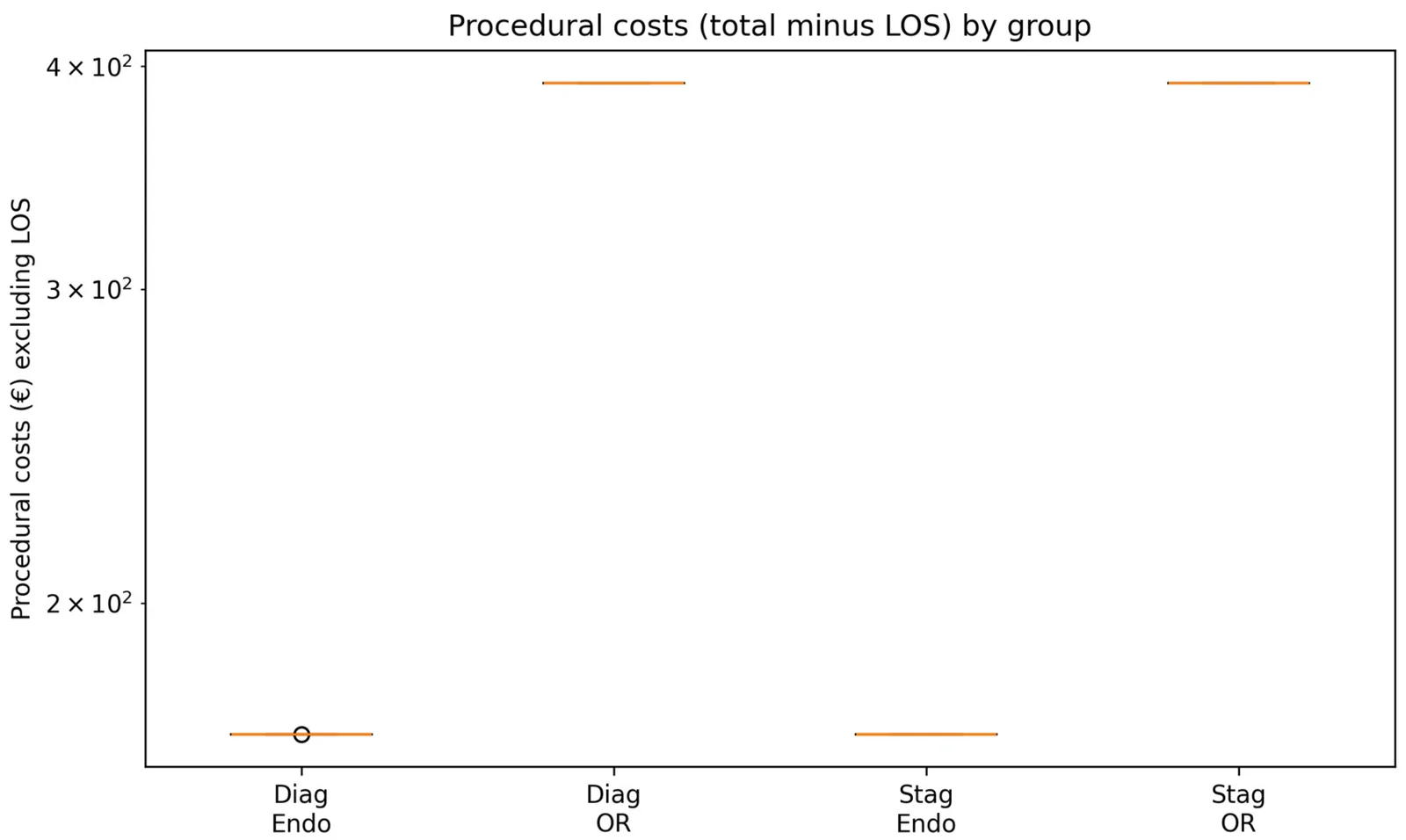
Procedural costs excluding length-of-stay (LOS) costs (total minus LOS component) by procedure type and procedural setting. Operating room procedures remained more costly than endoscopy suite procedures overall (Mann–Whitney U: *p* < 0.0001) and within diagnostic (*p* < 0.0001) and staging pathways (*p* < 0.0001). Between procedure types, non-LOS costs did not differ significantly within either the operating room (*p* = 0.117) or the endoscopy suite (*p* = 0.563). Abbreviations: Diag, diagnostic EBUS; Endo, endoscopy suite; Stag, staging EBUS; OR, operating room.

When isolating the non-hospitalization component (total minus LOS-related costs), the operating room setting remained associated with higher procedural costs (Table 4 and Figure 3).

**Figure 4:**
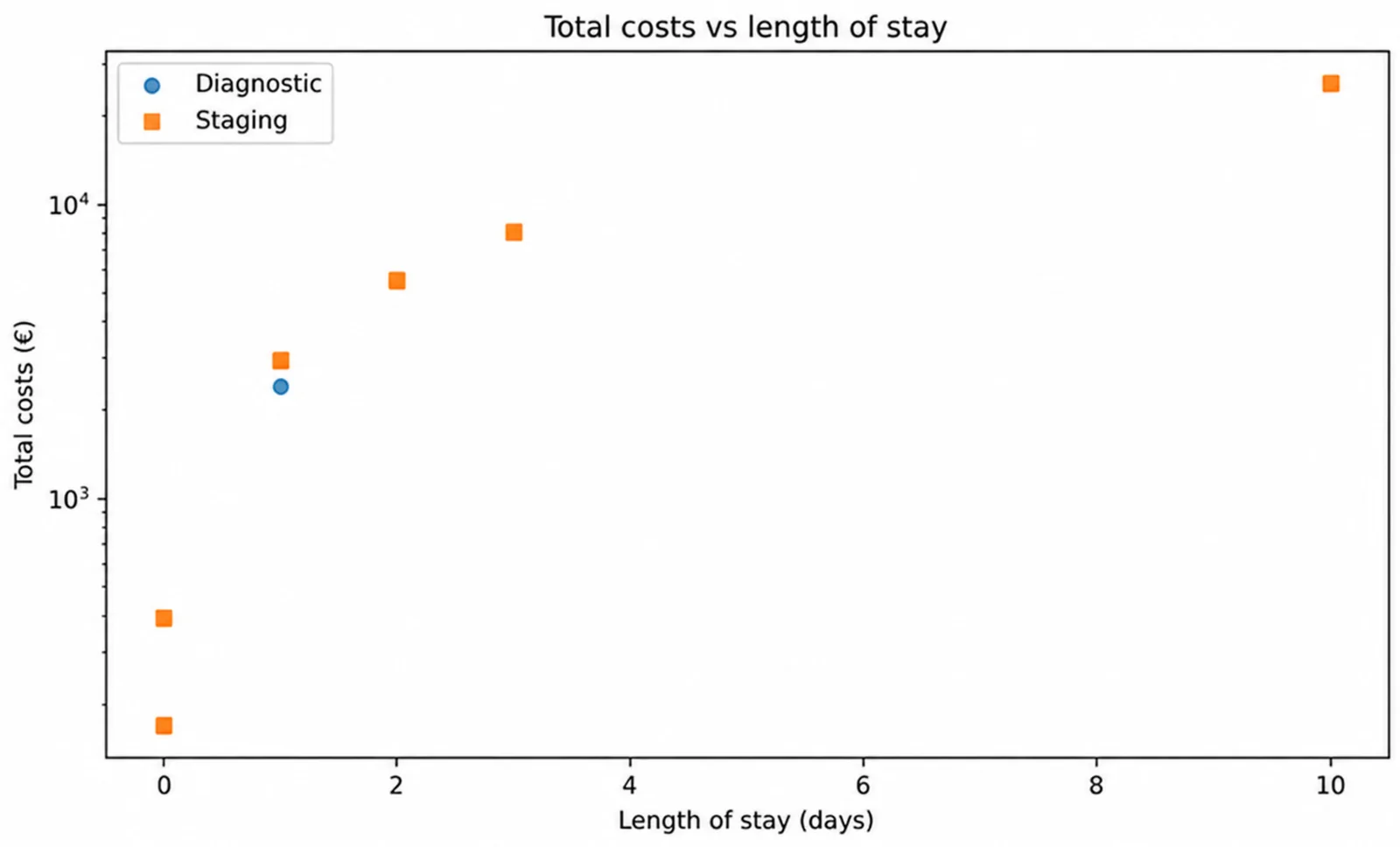
Relationship between total costs and length of stay. Scatterplot of total costs (log scale) versus LOS, stratified by procedure type. The strong positive association indicates that hospitalization duration is the dominant contributor to overall costs (see Table 4 and Table 6). Abbreviations: Diag, diagnostic EBUS; Endo, endoscopy suite; Stag, staging EBUS; OR, operating room.

Among hospitalized cases (LOS > 0), the LOS-related component accounted for the majority of total costs (Table 4), and the relationship between LOS and total costs is illustrated in Figure 4.

In multivariable models including procedural setting, procedure type, their interaction, and LOS, the latter emerged as the dominant cost driver, and the operating room setting remained associated with higher costs even after accounting for LOS; the interaction term suggested, at most, a marginal additional contribution (Table 6).

Findings were consistent in the additive sensitivity model (Table S11). Notably, the operating room coefficient should be interpreted as the incremental non-LOS (facility- or anesthesia-related) cost component attributable to the procedural setting at a given LOS, rather than as the unadjusted difference in total costs. Cost findings should also be interpreted in conjunction with cytology-based yield, sample adequacy, and the limited ascertainment of adverse events described above, rather than as a stand-alone argument for universal outpatient management.

Because systematic diagnostic verification remained incomplete and selectively available, comprehensive adverse event capture and patient-centered outcomes were unavailable, and downstream diagnostic investigations, repeat invasive procedures, subsequent healthcare utilization, and time to definitive diagnosis were not captured, these findings should be interpreted as an analysis of immediate pathway-related resource utilization rather than as a formal cost-effectiveness analysis.

## 4. Discussion

Endobronchial ultrasound-guided transbronchial needle aspiration (EBUS-TBNA) is a highly accurate, minimally invasive procedure recommended as a first-line invasive approach for nodal staging in non-small-cell lung cancer within contemporary evidence-based algorithms [1,2,23]. Randomized trials and health technology assessments have shown that endosonography-based strategies can achieve staging performance comparable to surgical staging pathways while reducing invasiveness and, in many systems, overall resource use [10,19,20,24]. In this single-center observational study, two institutional EBUS-TBNA care pathways showed similar cytology-based yield in the diagnostic cohort and similar sample adequacy in the staging cohort, while differing substantially in length of stay and costs. The main contribution of the study is therefore not the demonstration of superiority or equivalence of one anesthesia modality over another, but the real-world evaluation of resource utilization associated with outpatient endoscopy suite versus operating room EBUS-TBNA pathways.

In this sense, the present findings contribute to a practical institutional question: whether EBUS-TBNA should routinely depend on an operating room pathway or whether outpatient endoscopy suite delivery may represent a viable alternative for appropriately selected patients, while operating room resources remain available when anesthesiology-led airway management, higher monitoring intensity, or combined/technically complex procedures are required.

The results should be interpreted as a comparison between two care pathways rather than as an isolated comparison between sedation and general anesthesia. The outpatient and operating room pathways differed in procedural setting, airway and ventilation strategy, monitoring environment, recovery logistics, and hospitalization policy. Moreover, allocation was nonrandom and was influenced by organizational availability, anticipated procedural complexity, airway/anesthetic considerations, patient-level clinical factors, and consultant judgment. Therefore, confounding by indication is likely, and the observed associations should not be interpreted causally.

Among the available cytology-based endpoints, the lack of a meaningful difference in yield between pathways is consistent with previous randomized, prospective, and retrospective studies reporting broadly similar diagnostic yields across anesthesia strategies when sampling techniques and workflows are standardized [8,9,10,11,12,25]. However, the present study cannot determine the independent effect of the depth of anesthesia or the procedural setting on diagnostic accuracy.

Systematic diagnostic verification through clinical follow-up, repeat biopsy, imaging follow-up, or final diagnosis adjudication was not available for the entire cohort. However, postoperative nodal pathology could be retrospectively retrieved in 60 patients from the staging cohort, providing an exploratory surgical reference in a selected subset. Among the 58 patients with interpretable EBUS-TBNA results, patient-level N-stage concordance was 75.9%, while postoperative nodal upstaging occurred in 19.0%. Importantly, seven of the 11 upstaging cases involved nodal stations that had not been sampled during the index EBUS-TBNA, whereas only four involved a previously sampled negative station subsequently found to be positive at surgery.

These findings are consistent with previous surgical validation series showing that postoperative nodal upstaging after EBUS-TBNA may reflect both true station-matched false negative sampling and nodal disease in stations that were not sampled or were outside the routine reach of the EBUS examination [26,27,28].

Because this surgical verification was selective and unavailable for most of the staging cohort, these findings provide only partial postoperative validation and do not permit estimation of sensitivity, specificity, negative predictive value, false negative rate, or overall diagnostic accuracy.

Patients who did not proceed to surgery because of disease extent, comorbidity, treatment choice or personal preference, as well as cases for which postoperative records could not be retrieved, were necessarily absent from this verification subset. Therefore, substantial verification bias remains.

Cytology-based yield after EBUS-TBNA is influenced by multiple factors beyond the anesthesia strategy, including operator experience, lymph node location and size, sampling strategy, number of passes, needle type, tissue processing, ROSE availability, and the underlying disease spectrum [22,29,30,31,32]. In the present study, several technical factors were standardized across pathways: all procedures were performed within the same institutional unit under senior thoracic surgery supervision, using the same echo-bronchoscope platform, ultrasound system, 22G EBUS needle, and a protocol-mandated minimum of three passes per nodal station sampled. These factors may have reduced technical heterogeneity between pathways. However, lymph node location, clinical indication, ROSE availability, and case complexity may still have influenced cytology-based yield and cannot be fully disentangled from pathway allocation in this retrospective design.

The shorter recorded procedure duration in the operating room pathway may reflect a more controlled anesthetic environment, including cough suppression, stable airway access via a laryngeal mask airway, immobility, and continuous anesthesiology support. However, this interpretation remains speculative. Procedure duration was not uniformly recorded, and completeness was substantially higher in operating room cases than in outpatient procedures. Moreover, recorded duration may have captured different workflow intervals across settings. Therefore, duration findings should be interpreted as exploratory and cannot be used to infer that general anesthesia independently shortened EBUS-TBNA time.

ROSE utilization also requires cautious interpretation. In this dataset, ROSE relied on the Pathology Service’s scheduling and availability rather than on random allocation. Because ROSE may influence adequacy assessment, number of passes, and procedural decision-making, its variable availability represents a potential source of procedural confounding [29,30,31]. Similarly, sedation in the outpatient pathway was titrated according to institutional practice, but sedation depth scores were not systematically recorded as analyzable case-level variables; therefore, the study cannot evaluate whether within-pathway variations in sedation depth influenced cough, tolerance, sampling intensity, duration, or cytology-based yield.

The economic findings should be interpreted together with cytology-based yield and documented adverse events [14,19,20,21]. In this cohort, the outpatient pathway was associated with substantially lower LOS-driven and non-LOS procedural costs. In contrast, cytology-based yield in the diagnostic cohort and sample adequacy in the staging cohort were not materially lower than in the operating room pathway. However, because systematic diagnostic verification remained incomplete and selectively available, systematic post-procedural follow-up was unavailable, and adverse event capture was incomplete, cost advantage alone should not be interpreted as sufficient justification for universal outpatient management.

### Institutional Implications

From an institutional perspective, these findings support the prospective development and evaluation of structured pathway allocation criteria. In current practice, outpatient endoscopy suite EBUS-TBNA may be considered for appropriately selected patients without anticipated airway, anesthetic, monitoring, or technical complexity, whereas the operating room pathway should remain available when anesthesiology-led airway management, higher-intensity monitoring, combined procedures, or technically complex sampling are anticipated. These considerations represent pragmatic institutional allocation principles rather than validated patient selection criteria, as the present retrospective dataset does not permit derivation of specific clinical thresholds or a formal risk model. At our institution, these findings may inform the prospective development of explicit triage criteria and standardized documentation of sedation depth, ROSE availability, number of needle passes, adverse events, conversion or escalation, post-procedural outcomes, diagnostic verification, and resource utilization. The aim is not to eliminate anesthesiology-supported or operating room EBUS-TBNA, but to evaluate whether a structured outpatient pathway can reduce default operating room dependence in appropriately selected patients.

The main limitation of this study is its single-center, nonrandomized observational design. Allocation to the outpatient or operating room pathway may have been influenced by physician preference, organizational constraints, patient characteristics, anticipated procedural complexity, and airway/anesthetic considerations, resulting in residual confounding by indication. Several clinically relevant selection and procedural variables were not systematically available in the retrospective dataset, including ASA physical status, which has been investigated as a potential risk stratification variable in EBUS-TBNA [33], BMI, COPD or obstructive sleep apnea, baseline oxygen requirement, anticoagulant or antiplatelet therapy, lesion or lymph node size and location, detailed PET characteristics beyond available PET documentation, prior nondiagnostic bronchoscopy, need for additional concurrent procedures, sedation depth scores, total lidocaine dose, and minor or patient-reported procedural events such as cough, transient desaturation not requiring escalation, discomfort, and post-discharge symptoms. Therefore, although available variables related to indication, diagnostic suspicion, nodal stations sampled, PET documentation, and ROSE utilization were reported, adjustment for selection mechanisms remained incomplete. Moreover, because key case-level selection variables were not systematically available, propensity score matching or inverse probability weighting was deemed methodologically unreliable and therefore not performed. In addition, postoperative pathological verification was available only in a selected surgical subset. The probability of proceeding to surgery and of having retrievable postoperative pathology was influenced by disease extent, comorbidity, treatment choice, patient preference, and record availability; therefore, this additional analysis is also subject to verification and selection bias.

A combined EBUS/EUS strategy was not part of the institutional pathway evaluated in this retrospective dataset. Therefore, the present study cannot determine whether the addition of transesophageal endosonography would have increased nodal coverage or reduced postoperative upstaging. This consideration is relevant because several upstaging cases involved stations that had not been sampled during the index EBUS-TBNA, including stations 5 and/or 6, which were not routinely sampled through our institutional EBUS pathway [23,26,34,35].

Postoperative pathological verification was available only in a selected surgical subset and therefore remains subject to verification bias. Consequently, the exploratory postoperative analysis cannot provide sensitivity, specificity, negative predictive value, false negative rate, or overall diagnostic accuracy for either pathway.

Procedure duration was not uniformly captured across pathways and was analyzed using complete cases only; therefore, duration analyses should be considered exploratory. ROSE availability was not randomized and depended on Pathology Service scheduling. Sedation depth scores were not systematically recorded as analyzable case-level variables. Finally, adverse event ascertainment was retrospective and based on procedural documentation; this is particularly relevant because respiratory and other procedural complications have been specifically evaluated in larger EBUS-TBNA series and registry-based studies [36,37]. Minor adverse events, delayed complications, post-discharge events, and patient-reported outcomes were not systematically collected. As a result, the study cannot provide a comprehensive comparative safety assessment [36,37].

Accordingly, the absence of documented prespecified intraprocedural adverse events should not be interpreted as evidence of absence of all peri-procedural complications or of equivalent safety between pathways. In particular, post-procedural respiratory failure was not systematically captured as a longitudinal study endpoint.

Overall, our findings suggest that, within a regulated outpatient endoscopy environment and a standardized sampling protocol, the outpatient EBUS-TBNA pathway was associated with lower LOS-driven and non-LOS procedural costs, while no material reduction in cytology-based yield was observed in appropriately selected patients. This interpretation remains conditional on the limitations of nonrandom allocation, incomplete variable selection, selective and incomplete diagnostic verification, and limited ascertainment of adverse events [10,14,19,20,21].

## 5. Conclusions

In this single-center observational comparison of two institutional EBUS-TBNA pathways, outpatient endoscopy suite procedures under sedation and operating room procedures under general anesthesia showed similar cytology-based diagnostic yield in the diagnostic cohort and similar sample adequacy in the staging cohort. Systematic diagnostic verification was not available for the entire cohort, although exploratory postoperative nodal pathology ascertainment provided partial validation in a selected subgroup of staging patients. Because diagnostic verification was incomplete and adverse event ascertainment was limited, neither diagnostic accuracy nor comparative safety can be inferred.

Because pathway allocation was nonrandom and reflected clinical, anesthetic, organizational, and consultant-level factors, residual confounding by indication is likely. The most robust finding is the substantial difference in resource utilization, with higher hospitalization and costs in the operating room pathway. These findings support the prospective development and evaluation of explicit institutional triage criteria for EBUS-TBNA pathway allocation, while preserving OR-based general anesthesia pathways for patients with anticipated airway, anesthetic, monitoring, or technical complexity.

Prospective multicenter studies with predefined allocation criteria, standardized follow-up, final diagnosis adjudication, comprehensive adverse event reporting, patient-centered outcomes, and complete capture of selection-related variables are warranted to confirm generalizability and identify subgroups who may benefit from outpatient versus OR-based EBUS-TBNA pathways [1,2,16,23].

## Figures and Tables

**Table 1:** Baseline characteristics and procedural metrics by cohort and procedural setting.

Cohort	Setting	N	Age, Years (Median [IQR])	Male, *n*/N (%)	LOS, Days (Median [IQR])	Duration, Min (Median [IQR])	Duration Available, *n*/N (%)
Diagnostic EBUS	Endoscopy suite	229.0	67.0 [57.1–73.8]	159/229 (69.4%)	0.0 [0.0–0.0]	65.0 [55.0–75.0]	117/229 (51.1%)
	Operating room	90.0	66.5 [57.9–73.8]	53/90 (58.9%)	1.0 [1.0–2.0]	25.0 [20.0–30.0]	86/90 (95.6%)
	*p*-value		0.779		<0.0001	<0.0001	
Staging EBUS	Endoscopy suite	85.0	71.7 [63.6–76.1]	50/85 (58.8%)	0.0 [0.0–0.0]	65.0 [52.5–75.0]	47/85 (55.3%)
	Operating room	42.0	70.4 [65.7–76.5]	27/42 (64.3%)	1.5 [1.0–2.0]	30.0 [20.0–35.0]	39/42 (92.9%)
	*p*-value		0.937		<0.0001	<0.0001	

Notes: Continuous variables are reported as median [IQR]. Sex and duration availability are reported as *n*/N (%). *p*-values compare settings within each cohort. Abbreviations: IQR, interquartile range; LOS, length of stay.

**Table 2:** Nodal stations sampled and, among biopsied nodes, PET positivity and ROSE utilization.

Cohort	Station	Biopsied (*n*)	PET+ Among Biopsied (Yes/Known)	PET Missing Among Biopsied	ROSE Yes Among Biopsied (Yes/Known)
Diagnostic EBUS	2L	1		1	1/1
	2R	1	1/1	0	1/1
	4L	10	5/6	4	9/10
	4R	104	51/55	49	55/104
	7	171	91/106	65	131/171
	10R	41	19/20	21	26/41
	10L	7	6/6	1	6/7
	11L	10	7/8	2	9/10
	11R	17	12/14	3	15/17
Staging EBUS	2L	0		0	
	2R	0		0	
	4L	14	5/14	0	9/14
	4R	47	15/44	3	35/47
	7	101	27/96	5	74/101
	10R	31	21/26	5	19/31
	10L	13	7/12	1	9/13
	11L	10	3/9	1	7/10
	11R	8	7/8	0	5/8

Notes: PET+ among biopsied nodes is reported as PET-positive/PET-known. ROSE: yes; among biopsied nodes, is reported as ROSE-performed/ROSE-known. NA indicates not applicable. Abbreviations: PET, positron emission tomography; ROSE, rapid on-site evaluation.

**Table 3:** Key clinical outcomes by cohort and procedural setting.

Cohort	Outcome	Endoscopy Suite	Operating Room	Effect (OR, 95% CI)	Effect (RR, 95% CI)	*p*-Value
Diagnostic EBUS	Diagnostic yield (binary)	108/229 (47.2%)	45/90 (50.0%)	1.12 (0.69–1.82)	1.06 (0.83–1.36)	0.740
	Suspicion–cytology concordance (exact match)	94/213 (44.1%)	33/82 (40.2%)	0.85	—	0.636
	Cohen’s κ (4-category agreement)	0.233	0.146	—	—	—
Staging EBUS	Cytology categories (negative/positive/not adequate)	65/85; 18/85; 2/85	31/42; 8/42; 3/42	—	—	0.422
	Sample adequacy (negative/positive vs. not adequate)	83/85 (97.6%)	39/42 (92.9%)	0.31 (0.05–1.95)	—	0.331
	Positivity among adequate samples	18/83 (21.7%)	8/39 (20.5%)	0.93 (0.37–2.38)	—	1.000

Notes: Effect sizes are reported as odds ratio (OR) with 95% confidence interval (CI); risk ratio (RR) is provided where applicable. Abbreviations: CI, confidence interval; OR, odds ratio; RR, risk ratio.

**Table 4:** Total costs and length of stay stratified by procedure type and procedural setting.

Procedure Type	Setting	N	Total Cost (€), Median [IQR]	Total Cost (€), Mean ± SD	LOS (Days), Median [IQR]	LOS > 0, *n* (%)	LOS Cost Share * (Median)
Diagnostic EBUS	Endoscopy suite	272	168.91 [168.91–168.91]	177.12 ± 135.43	0.0 [0.0–0.0]	1/272 (0.4%)	0.0
	Operating room	112	2943.11 [2943.11–5494.70]	3284.84 ± 1982.25	1.0 [1.0–2.0]	87/112 (77.7%)	0.867
Staging EBUS	Endoscopy suite	93	168.91 [168.91–168.91]	168.91 ± 0.00	0.0 [0.0–0.0]	0/93 (0.0%)	0.0
	Operating room	56	2943.11 [2943.11–5494.70]	4492.29 ± 3324.32	1.0 [1.0–2.0]	54/56 (96.4%)	0.867

Notes: Costs are expressed in euros (€). Continuous variables are reported as median [IQR] and mean ± SD. * LOS cost share indicates the fraction of total costs attributable to LOS costs (LOS costs (total)/total costs). Abbreviations: IQR, interquartile range; LOS, length of stay.

**Table 5:** Pairwise comparisons of total costs and LOS.

Variable	Comparison	Group A, Median [IQR]	Group B, Median [IQR]	*p*-Value
Total cost (€)	Operating room vs. Endoscopy suite (overall)	2943.11 [2943.11–5494.70]	168.91 [168.91–168.91]	<0.0001
	Staging vs. Diagnostic (overall)	168.91 [168.91–2943.11]	168.91 [168.91–391.52]	0.019
	Operating room vs. Endoscopy suite (diagnostic)	2943.11 [2943.11–5494.70]	168.91 [168.91–168.91]	<0.0001
	Operating room vs. Endoscopy suite (staging EBUS)	2943.11 [2943.11–5494.70]	168.91 [168.91–168.91]	<0.0001
	Staging EBUS vs. Diagnostic EBUS (operating room)	2943.11 [2943.11–5494.70]	2943.11 [2943.11–5494.70]	0.008
	Staging EBUS vs. Diagnostic EBUS (endoscopy suite)	168.91 [168.91–168.91]	168.91 [168.91–168.91]	0.563
LOS (days)	Staging EBUS vs. Diagnostic EBUS (operating room)	1.00 [1.00–2.00]	1.00 [1.00–2.00]	0.008

Notes: *p*-values are from two-sided Mann–Whitney U tests. Abbreviations: IQR, interquartile range; LOS, length of stay.

**Table 6:** Multivariable model for total costs including LOS (Gamma generalized linear model with log link).

Predictor	Cost Ratio (expβ)	95% CI	*p*-Value
Operating room (vs. endoscopy suite)	4.81	4.48–5.18	<0.0001
Staging EBUS (vs. diagnostic EBUS)	0.98	0.93–1.05	0.611
Interaction (operating room × staging EBUS)	1.10	1.00–1.23	0.062
LOS (per +1 day)	2.66	2.56–2.77	<0.0001

Notes: Cost ratios are interpreted multiplicatively (expβ). The model includes procedural setting, procedure type, their interaction, and LOS (days). Abbreviations: CI, confidence interval; LOS, length of stay.

## Data Availability

The datasets generated and analyzed during the current study are not publicly available due to institutional data protection policies and European privacy regulations (EU GDPR 2016/679), which restrict the sharing of sensitive health-related information. Aggregate data may be available upon reasonable request from the corresponding author, subject to ethical approval and compliance with data protection rules.

## Supplementary Materials

The following supporting information can be downloaded at: https://www.mdpi.com/article/10.3390/diagnostics16172767/s1, Table S1: ROSE utilization by cohort and procedural setting; Table S2: Sampling patterns of nodal stations and not adequate rates in the staging cohort; Table S3: Cytology adequacy by number of sampled nodal stations in the staging cohort; Table S4: Univariate association between number of sampled stations and not adequate cytology in the staging cohort; Table S5: Diagnostic yield and nondiagnostic categories by diagnostic suspicion group (diagnostic cohort); Table S6: Distribution of diagnostic suspicion and cytologic diagnoses in the diagnostic cohort; Table S7: Diagnostic suspicion distribution by procedural setting in the diagnostic cohort; Table S8: Distribution of baseline diagnosis and cytology results in staging cohort; Table S9: Association between sampled nodal station and cytology adequacy in the staging cohort; Table S10: Exploratory postoperative nodal pathology ascertainment in the staging EBUS-TBNA cohort; Table S11: Sensitivity analysis: additive model for total costs including LOS (linear regression with robust standard errors). Available supplementary analyses should be interpreted as exploratory and descriptive in relation to pathway selection, ROSE availability, sampling patterns, and missingness. Figure S1: Representative cytomorphological and immunocytochemical findings from an EBUS-TBNA sample diagnosed as small-cell lung carcinoma.

## Abbreviations

The following abbreviations are used in this manuscript:

18F-FDG	Fluorine-18 fluorodeoxyglucose
PET	Positron emission tomography
ADC	Adenocarcinoma
CI	Confidence interval
CT	Computed tomography
EBUS	Endobronchial ultrasound
EBUS-TBNA	Endobronchial ultrasound-guided transbronchial needle aspiration
ECG	Electrocardiogram
GA	General anesthesia
IQR	Interquartile range
LMA	Laryngeal mask airway
LOS	Length of stay
OR	Operating room
ROSE	Rapid on-site evaluation
RR	Risk ratio
SCC	Squamous cell carcinoma
SD	Standard deviation
TBNA	Transbronchial needle aspiration
TCI	Target-controlled infusion
TIVA	Total intravenous anesthesia
22G	22-Gauge
