# Supplementary material for: Beyond the Operating Room? Cytology-Based Yield and Resource Utilization of Outpatient Versus Operating Room EBUS-TBNA Pathways
Source: Diagnostics (Basel). 2026 Aug 28;16(17):2767. doi: 10.3390/diagnostics16172767 (PMC13565534; doi:10.3390/diagnostics16172767)
Supplement: Supplementary file 1 [file diagnostics-16-02767-s001.zip › diagnostics-4478861-supplementary.pdf]

Supplementary Tables

Table S1. ROSE utilization by cohort and procedural setting

Cohort	Setting	Procedures, *n*	Procedures with recorded sampled station, *n*	Procedure-level ROSE, *n/N* (%)	p-value*	Sampled nodal stations, *n*	Station-level ROSE, *n/N* (%)
Diagnostic EBUS	Endoscopic room	229	228	161/228 (70.6%)	0.891	255	177/255 (69.4%)
Diagnostic EBUS	Operating room	90	89	64/89 (71.9%)		107	76/107 (71.0%)
Staging EBUS	Endoscopic room	85	85	62/85 (72.9%)	1.000	146	102/146 (69.9%)
Staging EBUS	Operating room	42	42	30/42 (71.4%)		76	54/76 (71.1%)

* p-values compare endoscopic-room versus operating-room pathways within each cohort using Fisher's exact test at the procedure level.

Table S2. Sampling patterns of nodal stations and not-adequate rates in the staging cohort.

Pattern	Procedures, *n*	%	Not adequate, *n*	Not adequate %
7	37.0	28.9	1.0	2.7
4R+7	21.0	16.4	1.0	4.8
7+10R	9.0	7.0	0.0	0
10R	7.0	5.5	1.0	14.3
4R	7.0	5.5	0.0	0
4R+7+10R	6.0	4.7	0.0	0
7+10L	4.0	3.1	0.0	0
11L	4.0	3.1	1.0	25
7+11R	4.0	3.1	0.0	0
4L+7	4.0	3.1	1.0	25
Other patterns (n<4)	25.0	19.5	0.0	0
Test with p-value; OR (single-station vs multi-station)				Fisher p=0.665; OR=1.74

Notes: Patterns represent combinations of sampled stations per procedure. Only patterns with n≥4 are listed explicitly; remaining patterns are grouped. NA% denotes the proportion of not-adequate cytology within each pattern.

Table S3. Cytology adequacy by number of sampled nodal stations in the staging cohort.

Stations sampled (*n*)	Procedures, *n*	Not adequate, *n* (%)	Adequate, *n* (%)	Positive, *n* (%)	Negative, *n* (%)
1	60.0	3 (5.0%)	57 (95.0%)	9 (15.0%)	48 (80.0%)
2	46.0	2 (4.3%)	44 (95.7%)	14 (30.4%)	30 (65.2%)
3	17.0	0 (0.0%)	17 (100.0%)	4 (23.5%)	13 (76.5%)
4	4.0	0 (0.0%)	4 (100.0%)	0 (0.0%)	4 (100.0%)
5	1.0	0 (0.0%)	1 (100.0%)	0 (0.0%)	1 (100.0%)
Test with p-value		Mann–Whitney (NA vs Adequate): p=0.396	Fisher (1 vs ≥2): p=0.665	Fisher (≥3 vs <3): p=0.587	

Notes: Not-adequate rate is shown by number of stations sampled. p-values summarize nonparametric and Fisher exact comparisons. Interpretation is limited by low number of not-adequate events (n=5).

Table S4. Univariate association between number of sampled stations and not-adequate cytology in the staging cohort.

Predictor	Odds ratio for not adequate	p-value	Note
Stations sampled (per +1 station)	0.52 (0.13–2.13)	0.361	Univariate logistic regression; interpret cautiously due to low not-adequate event count (n=5).

Notes: Univariate logistic regression estimating the association between the number of sampled stations and the odds of a not-adequate result; interpret cautiously due to low event count.

Table S5. Diagnostic yield and non-diagnostic categories by diagnostic suspicion group (diagnostic cohort).

Diagnostic suspicion	*n*	Diagnostic (CR=1), n (%)	Negative (adequate, no suspicious cells), *n* (%)	Not adequate (insufficient material), *n* (%)	Diagnostic yield, % (95% CI)	p-value
Cancer suspicion	230	103 (44.8%)	122 (53.0%)	5 (2.2%)	44.8 (38.5–51.2)	
Sarcoidosis suspicion	40	25 (62.5%)	12 (30.0%)	3 (7.5%)	62.5 (47.0–75.8)	
Lymphoproliferative suspicion	24	11 (45.8%)	13 (54.2%)	0 (0.0%)	45.8 (27.9–64.9)	
Missing/Other suspicion	25	14 (56.0%)	9 (36.0%)	2 (8.0%)	56.0 (37.1–73.3)	
Global comparison across groups					Chi-square test (3×2 yield table)	0.116
Pairwise comparisons (Holm-adjusted)					Cancer suspicion vs Sarcoidosis suspicion: p=0.123; Cancer suspicion vs Lymphoproliferative suspicion: p=1.0; Sarcoidosis suspicion vs Lymphoproliferative suspicion: p=0.415	—

Notes: Negative indicates adequate material with no suspicious cells; not adequate indicates insufficient material precluding a definitive diagnosis. Missing/Other suspicion includes cases coded as "Other" (suspicion=3) and cases with missing suspicion coding. Global p-value refers to chi-square test comparing diagnostic yield across the three suspicion groups.

Table S6. Distribution of diagnostic suspicion and cytologic diagnoses in the diagnostic cohort.

Domain	Category	*n*	%
Diagnostic suspicion	Cancer	230	72.1
	Sarcoidosis	40	12.5
	Lymphoproliferative disease	24	7.5
	Missing	24	7.5
	Other	1	0.3
Cytologic diagnosis	Negative	156	48.9
	Adenocarcinoma (ADC)	49	15.4
	Sarcoidosis	33	10.3
	Other primary lung cancer	26	8.2
	Squamous cell carcinoma (SCC)	16	5.0
	Metastasis	15	4.7
	Lymphoproliferative disease	14	4.4
	Not adequate	10	3.1

Notes: Percentages are calculated over the full diagnostic cohort (Diagnostic EBUS; N=319). Abbreviations: ADC, adenocarcinoma; SCC, squamous cell carcinoma.

Table S7. Diagnostic suspicion distribution by procedural setting in the diagnostic cohort.

Setting	*n*	Cancer	Lymphoproliferative disease	Sarcoidosis	Other
Endoscopic room	213	166 (77.9%)	21 (9.9%)	26 (12.2%)	0 (0.0%)
Operating room	82	64 (78.0%)	3 (3.7%)	14 (17.1%)	1 (1.2%)

Notes: Counts are shown with row percentages. Association between setting and diagnostic suspicion was assessed with a chi-square test (χ^2=6.43, df=3, p=0.092); effect size: Cramer's V=0.148. N analyzed=295.

Table S8. Distribution of baseline diagnosis and cytology results in staging cohort.

Domain	Category	*n*	%
Baseline diagnosis category	SCC	84	65.6
	ADC	31	24.2
	Other primary lung cancer	7	5.5
	Missing	3	2.3
	Pulmonary metastasis	3	2.3
Cytology result category	Negative	96	75.0
	Positive	27	21.1
	Not adequate	5	3.9

Notes: Percentages are calculated over the full staging cohort (Staging EBUS; N=128). Abbreviations: ADC, adenocarcinoma; SCC, squamous cell carcinoma.

Table S9. Association between sampled nodal station and cytology adequacy in the staging cohort.

Station	Biopsied *n*	Not biopsied *n*	Not adequate among biopsied	Not adequate among not biopsied	OR (NA)†	p-value
2L	0	128		5/128		
2R	0	128		5/128		
4L	14	114	1/14 (7.1%)	4/114 (3.5%)	2.12 (0.22–20.38)	0.445
4R	47	81	1/47 (2.1%)	4/81 (4.9%)	0.42 (0.05–3.86)	0.651
7	101	27	3/101 (3.0%)	2/27 (7.4%)	0.38 (0.06–2.41)	0.284
10L	13	115	0/13 (0.0%)	5/115 (4.3%)	0.74 (0.04–14.21)‡	1.0
10R	31	97	1/31 (3.2%)	4/97 (4.1%)	0.78 (0.08–7.20)	1.0
11L	10	118	1/10 (10.0%)	4/118 (3.4%)	3.17 (0.32–31.40)	0.339
11R	8	120	0/8 (0.0%)	5/120 (4.2%)	1.24 (0.06–24.26)‡	1.0

Notes: Outcome was dichotomized as adequate (negative or positive cytology) versus not adequate. Associations are procedure-level (station sampled yes/no vs cytology adequacy) and were tested using Fisher's exact test. †Odds ratio refers to the odds of a not-adequate result when the station was sampled versus not sampled. ‡Continuity correction (0.5) applied for confidence interval estimation due to zero cells. Interpretation is limited by the low number of not-adequate cases.

Table S10. Exploratory postoperative nodal-pathology ascertainment in the staging EBUS-TBNA cohort

**Panel A – Patient-level ascertainment**	
Outcome	n/N (%)
Total staging EBUS-TBNA cohort	128
Postoperative pathological nodal stage available	60/128 (46.9%)
Interpretable EBUS-TBNA among cases with postoperative pathological nodal stage available	58/60 (96.7%)
EBUS/postoperative pathological N-stage concordant	44/58 (75.9%)
Postoperative nodal upstaging	11/58 (19.0%)
EBUS-positive followed by postoperative pathological N0	3/58 (5.2%)
Documented interval neoadjuvant treatment among EBUS-positive/postoperative pathological N0 cases	2/3 (66.7%)
**Panel B – Characterization of the 11 upstaging cases**	
Characterization	n/N (%)
Postoperative positive station not sampled during index EBUS-TBNA	7/11 (63.6%)
Same station sampled negative at EBUS-TBNA and positive at surgical pathology	4/11 (36.4%)

Notes: Two EBUS-positive cases with postoperative pathological N0 received neoadjuvant treatment after EBUS-TBNA and before surgery and were therefore not interpreted as false-positive EBUS-TBNA results.

Among the four station-matched discordances, one patient had already been identified as N1 by EBUS-TBNA but was subsequently found to have additional N2 involvement at a previously negative station 7; this case therefore represented nodal understaging rather than an overall false-negative EBUS-TBNA examination. Stations 5 and 6 were not routinely sampled through the institutional EBUS pathway.

Table S11. Sensitivity analysis: additive model for total costs including LOS (linear regression with robust standard errors).

Predictor	Incremental cost (€)	95% CI (€)	p-value
Operating room (vs endoscopic room)	226.00	218.49–233.51	<0.0001
Staging EBUS (vs diagnostic EBUS)	1.16	-1.12–3.44	0.318
Interaction (operating room × staging EBUS)	-0.23	-1.02–0.55	0.563
LOS (per +1 day)	2549.63	2544.69–2554.56	<0.0001

Notes: Sensitivity analysis using linear regression with robust standard errors. Estimates represent additive changes in total costs (€). Because LOS (days) is included in the model, the coefficient for procedural setting (operating room vs endoscopic room) should be interpreted as the incremental non-LOS cost component attributable to the setting (facility/anesthesia infrastructure) at a given LOS, rather than the unadjusted difference in total costs. Abbreviations: CI, confidence interval; LOS, length of stay.

Figure S1. Representative cytomorphological and immunocytochemical findings from an EBUS-TBNA sample diagnosed as small-cell lung carcinoma.

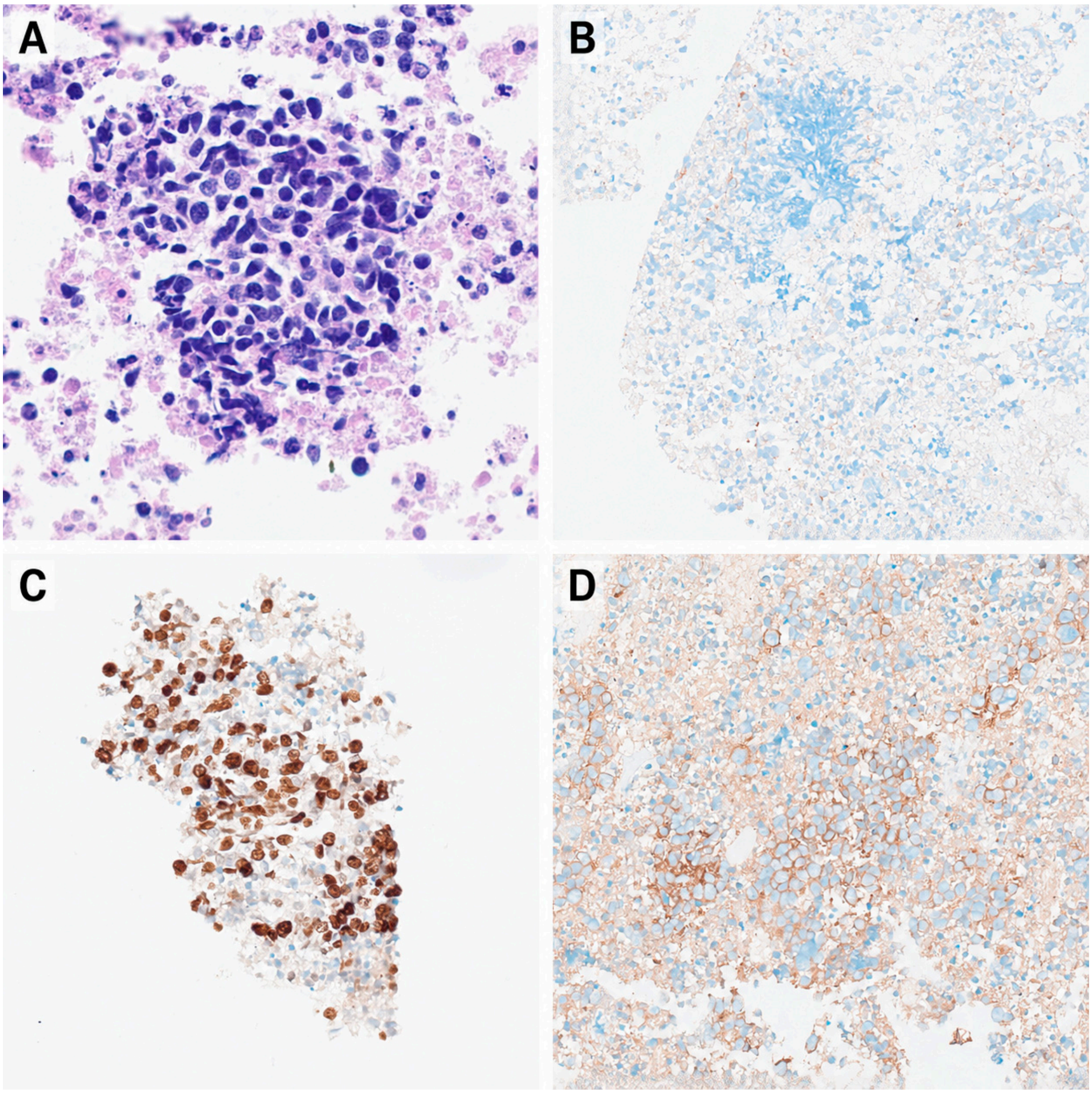

Notes: A) Hematoxylin and eosin/cytology preparation. High-power view of a small-cell lung cancer showing atypical small cells arranged in loose clusters. Note the high nuclear-to-cytoplasmic ratio, evident nuclear molding with crushing artifacts, and coarse "salt-and-pepper" chromatin pattern. (B) Immunohistochemical staining for Chromogranin A shows focal granular cytoplasmic positivity within the malignant cell clusters. This stain, together with the diffuse positivity of synaptophysin (D), confirms the neuroendocrine nature of the tumor; (C) Ki-67-stained cell block section revealing a very high proliferation index (>80%), consistent with a malignant neuroendocrine lesion. (Unit of Anatomic Pathology, Azienda di Rilievo Nazionale ed Alta Specializzazione "G. Brotzu", Piazza A. Ricchi 1, 09121 Cagliari, Italy)
